# Different Treatments for Sugarcane Juice Preservation

**DOI:** 10.3390/foods12020311

**Published:** 2023-01-09

**Authors:** Pooja Dhansu, Bakshi Ram, Ashish Kumar Singh, Sudhir Kumar Tomar, Ramaiyan Karuppaiyan, Ravinder Kumar, Manohar Lal Chhabra, Ajay Singh, Arun Kumar Raja, Prashant Kaushik, Shashi Kant Pandey

**Affiliations:** 1 ICAR–Sugarcane Breeding Institute, Regional Center, Karnal 132001, Haryana, India; raviagricos@gmail.com (R.K.); mlchhabrasbik@gmail.com (M.L.C.); s_kantpandey@yahoo.com (S.K.P.); 2 ICAR–Sugarcane Breeding Institute, Coimbatore 641007, Tamil Nadu, India; bryadav2003@yahoo.com (B.R.); sikkimkarups@gmail.com (R.K.); arunkr.plphy@gmail.com (A.K.R.); 3 ICAR–National Dairy Research Institute, Karnal 132001, Haryana, India; aksndri@gmail.com (A.K.S.); sudhirndri@gmail.com (S.K.T.); 4 Department of Food Technology, Mata Gujri College, Fatehgarh Sahib 140406, Punjab, India; ajay3singh@gmail.com; 5 Instituto de Conservación y Mejora de la Agrodiversidad Valenciana, Universitat Politècnica de València, 46022 Valencia, Spain; prakau@doctor.upv.es

**Keywords:** sulphitation, acidification, steam, bottled juice, preservation, sensory, sugarcane

## Abstract

This investigation aimed to optimize the time, pH, pressure, and temperature of sugarcane juice pasteurization and to develop a “ready to serve” bottled sugarcane juice with a high preservation efficiency. Fresh sugarcane juice was extracted from sugarcane genotype Co 89003, and beverage samples were collected using three different treatments: sulphitation of juice with the addition of potassium metabisulphite (KMS-25, 50, 100, and 150 ppm), acidification of juice (addition of citric acid, to reduce the pH of the juice to 4.8, 4.5, and 4.25), and steam treatment of the canes (5 min, 10, and 15 min at 7 psi). In all treatments, the juice was pasteurized in glass bottles @ 65 °C for 25 min and stored at low temperature (5 °C) in pre-sterilized glass bottles. Juice properties such as the ˚Brix, total sugar, pH, and total phenolic content decreased with storage, whereas the microbial count, titrable acidity, and reducing sugar content significantly increased during storage. The addition of KMS, citric acid, and the steam treatment reduced the browning of juice and maintained the color of juice during storage, by inhibiting the polyphenol oxidase enzyme activity, from 0.571 unit/mL to 0.1 unit/mL. Among the selected treatments, sugarcane juice with KMS (100 and 150 ppm) and steam treatment of the canes for 5 and 10 min at 7 psi showed the minimum changes in physico-chemical properties, sensory qualities, and restricted microbial growth. Thesulphitation treatment with pasteurization proved best for increasing the shelf life of sugarcane juice upto 90 days with refrigeration. Similarly, the steam-subjected cane juice (10 and 15 min at 7 psi) could be effectively preserved for upto 30 days with refrigeration, without any preservative.

## 1. Introduction

Sugarcane (*Saccharum* ssp.) belongs to the family Poaceae, ranks among the ten most planted crops in the world, and is widely distributed in tropical and subtropical regions of the world [1,2]. Fresh sugarcane (*Saccharum officinarum* L.) juice is well-liked in various countries, with the highest preference particularly in Asian countries: India, China, Malaysia, and Thailand. Easy access, cheap price, sweetish taste, and beneficial impact on health are the reasons that govern its selection. Sugarcane juice is high in sucrose, polyphenolic compounds, minerals (such as potassium, calcium, salt, iron, and magnesium), ascorbic acid, colors, and fine bagasse [3], which make it a remedy for many diseases such as jaundice, fever, and kidney problems, while also working to strengthen the kidneys, heart, eyes, and brain [4]. Sugarcane juice contains flavonoids and this protects cells from degenerative processes and reduces the development of cancer and cardiovascular disease [5]. In India, it is solely exploited for table sugar production through its juice. 

Food producers now seek natural, ecologically friendly, and safe food preservatives that are less expensive, more nourishing, and simple to obtain for the health-conscious consumer market [6,7,8]. Sugarcane juice is of high nutritional significance, but it experiences major changes in terms of its color, sedimentation, and preservation inefficiency [9]. To extend the shelf life of sugarcane juice, it is essential to develop adequate preservation technology. Various studies have considered a number of sugarcane juice preservation techniques, including chemical, thermal, and non-thermal methods. Development of effective treatments for sugarcane juice, which can maintain its quality, freshness, and nutrient content, could allowits wider marketing and consumption.Raw sugarcane juice is carbohydrate-rich, non-acidic (>5.0 pH), and susceptible to being attacked by yeasts, molds, and other spoilage pathogenic microflora [10]; thus, fresh extracted juice develops a brown color and tastes sour within a few hours of extraction when not stored appropriately, representing a potential health hazard to consumers [11]. Furthermore, browning, sedimentation defects during storage, and lack of hygienic practices while processing lower its sensory acceptability. The major problem associated with sugarcane juice production is polyphenol oxidase activity (PPO), which causes a change in the appearance and organoleptic properties [12]. Chemical and enzymatic inversions also affect its sensory profile [13]. Many food preservatives within the permissible range can be used to preserve fruit juice for longer periods. As per the Food Safety and Standards Regulation (Food Product Standard and Food Additives) of the Food Safety and Standard Authority of India (FSSAI) 2011 [14], the maximum limit of potassium metabisulphite (KMS) is 700 ppm for use as preservative in fruit juice and beverages. Potassium metabisulphite, citric acid, sodium benzoate, and ascorbic acid are generally recognized as safe (GRAS) by the Food and Drug Administration (FDA) and Food and Agriculture Organization of the United Nations [15]. Enzymatic browning of sugarcane juice can be controlled by thermal treatment and use of some preservatives such as ascorbic acid, citric acid, and potassium metabisulphite, because they inhibit the activity of polyphenol oxidase enzyme [12]. The addition of KMS can reduce the microbial activity in sugarcane juice and enhance its shelf life [16]. Potassium metabisulphite and citric acid help in the maintenance of titrable acidity and pH, restrict microbial activity, and preserved mango pulp up to 90 days [17]. Ascorbic acid is probably the most widely used inhibitor of enzyme activity, and in addition to its reducing properties, it also slightly lowers pH [18]. It is hard to store sugarcane juice without the addition of a preservative [19].

Due to the high nutritional value and cheap price of sugarcane juice, it is vitally important to provide raw juice to non-sugarcane growing areas of the country and to make it easily available everywhere; and hence, preservation of sugarcane juice is necessary. Therefore, there is an imperative requirement to develop a process for the preservation of sugarcane juice for longer periods via some modifications withdifferent technologies. Although several studies have previously documented the preservation of fruit juices, in general following a single protocol, information about preserving bottled sugarcane juice through different methods is limited. Steam treatment for 10 min followed by autoclaving at 126 °C and 121 °C for 10 min, and treatment with (0.1%) sodium metabisulfite enhanced the shelf life for 15 weeks at 4 °C [15]. Khare et al. [16] enhanced the shelf life of sugarcane juice by 60 days through a pasteurization process at 75 °C for 10 min, after addition of 3 mL lemon and 1 g salt, 0.6 mL ginger per 100 mL of sugarcane juice, and the addition of KMS at 225 ppm. In addition, Ali et al. [20] also reported that pasteurization at 90 °C for 5 min, with the addition of citric acid to maintain the pH at 4.3, enhanced the shelf life of sugarcane juice up to 120 days. Leistner and Gorris [21] observed that for preserving any food and beverage, there were physical, physicochemical, microbially derived, and miscellaneous hurdles. Of these, temperature, acidity, competitive microorganisms, and preservatives are the most important. Therefore, the appropriate and justified use of preservatives can provide better methods of juice preservation. Hence, the present study was conducted to optimize the process of the preservation of bottled sugarcane juice as ready-to-serve with a high consumer acceptability.

## 2. Materials and Methods

### 2.1. Plant Material and Pre-Processing Operations

Sugarcane variety Co 89003 was selected (on the basis of its softness, being easy to peel, and better juice extraction efficiency) for this study from the field of ICAR-Sugarcane Breeding Institute, Regional Centre, Karnal, India (longitude of 67°58′ North and latitude 29°43′ East). Fresh sugarcane plants were cleaned with a heavy flow of potable water, hand-peeled, and cut to a uniform length of about 40 cm. Canes were harvested at the 10 month crop age. After harvesting, mold- and insect-infested sugarcanes were discarded and good canes were cleaned and washed with a heavy flow of water to remove dirt, soil, and microorganisms. The handling area was cleaned with disinfectant and canes were peeled and washed with boiling water. Canes were crushed using a stainless steel crusher with a 65–70% extraction efficiency, which was sterilized with boiling water followed by 10 ppm sodium hypochlorite solution.

#### 2.1.1. Steam Treatment

Peeled canes were subjected to autoclaving for 5, 10, and 15 min at 7 psi, which served as test sample, whereas peeled canes without steaming served as a control. After cooling, canes were crushed to obtain sugarcane juice. The juice was filtered through multilayer muslin cloth, filled in sterilized glass bottles under aseptic conditions, and subsequently pasteurized for 25 min at 65 °C; after cooling at room temperature the juice bottles were stored ina refrigerator. Samples treated with steam were evaluated for their microbial, physico-chemical, and sensory characteristics at 0, 15, 30, and 60 days, since after 60 days, the juice had deteriorated.

#### 2.1.2. Sulphitation

Juice was directly extracted without subjecting the canes to steaming. Theextracted juice was filtered through an autoclave-sterilized muslin cloth. Filtered juice was prepared with theaddition of potassium metabisulphite (KMS), i.e., 25, 50, 100, and 150 ppm, and a control (without sulphitation). Juice was filled in sterilized glass bottles under aseptic conditions and pasteurized for 20 min at 65 °C. After attaining room temperature, the juice was stored in a refrigerator. Samples treated with sulphitation (KMS) were evaluated for their microbial, physico-chemical, and sensory characteristics at 0, 15, 30, 60, and 90 days.

#### 2.1.3. Acidification

Extracted juice was filtered through autoclave-sterilized muslin cloth, and the pH of the juice was lowered to 4.8, 4.5, and 4.25 with the addition of 50% citric acid solution and compared to thereference to juice (5.1 pH) labeled as a control. Juice was pouredinto sterilized glass bottles under aseptic conditions and subjected toin-bottle pasteurization (65 °C/25 min), followed bycooling to room temperature and refrigeration. Samples treated with acidification were tested for their microbial, physico-chemical, and sensory characteristics at 0, 15, 30, and 60 days.

### 2.2. Microbial Examination

During the different stages of storage, the sugarcane juice of all the treatments was analyzed for its microflora enumeration through a standard plate count (SPC), coliform count, and yeast and mold count. Selected decimal dilutions (10^−1^–10^−4^) for SPC were carried out with 0.85% NaCl solution; aliquots of one ml were spread on plate count agar and incubated at 37 °C/24–48 h. Coliform, yeast, and mold count decimal dilutions (10^−1^–10^−2^) of the juice were made and spread on to violet red bile agar (VRBA @ 37 °C/48 h) and potato dextrose agar (PDA 25 °C/72 h), respectively. The different counts were expressed as cfu/mL [22].

### 2.3. Physico-Chemical Characterization

Physico-chemical characteristics including ˚Brix value (%) measured by refractometer, pH by digital pH meter (cyber scan pH 510, Eutech), total sugar (%), reducing sugar (%), and titrable acidity (as % citric acid) were estimated as per the method of Ranganna [23]. SO_2_ (ppm) in sulphitated juice was measured using an optimized Monier–Williams (OMW) method.

#### 2.3.1. Total Phenolic Content

The Folin–Ciocalteu method, with some modifications, was used to measure the total polyphenolic content [24]. Aliquots of 0.5 mL of juice extract were mixed with 2.5 mL Folin–Ciocalteu reagent. A reagent blankwas prepared instead of a sample. After 5 min incubation at room temperature, 1 mL sodium carbonate solution (7.5%) was added. Samples were incubated at room temperature for 1 h in the dark, and the absorbance was measured at 765 nm against a blank. The total phenolic content was calculated from the calibration curve of gallic acid and expressed as gallic acid equivalents (GAE), in milligrams per gram of the sample.

#### 2.3.2. Polyphenol Oxidase Enzyme Activity (Unit/mL)

Polyphenol oxidase (PPO) was measured as suggested by Ozoglu and Bayindirli [25], and the reaction was started by adding 1 mL of 0.2 M catechol into the mixture containing 0.5 mL of sugarcane juice and 2 mL of 50 mM phosphate buffer (pH 6.5). The absorbance was recorded every 1 min at 420 nm. 

### 2.4. Sensory Evaluation

Treatment exposed juice was evaluated by means of sensory preferences, with reference to the control sample: color and appearance, flavor, and overall acceptability. Sensory judgment was madeby a semi-trained panel with 10 members with food science and microbiology concerns. Evaluations were carried out with anine-point hedonic score, where the samples were blindly presented to the panel in coded form [26].

### 2.5. Statistical Analysis

Analysis of variance (ANOVA) was performed using general linear model statement in SAS 9.3 software (SAS Institute, Cary, NC, USA) on the data obtained from all three treatments with three replications i.e., n = 3. Statistical differences were also computed among the means of treatment, storage time, and theirinteraction usingtwo way ANOVA; and Tukey’s test was conducted to display the differences among the means and storage time.

## 3. Results and Discussion

### 3.1. Optimization of Treatment

Freshly harvested sugarcane stalks were subjected to steam treatment for 5, 10, and 15 min at 7 psi. It was observed that the 10 and 15 min steam-heated samples followed by pasteurization achieved a satisfactory sensory score for appearance, flavor, and overall acceptability (Table 1). Parallel trials for Sulphitation with 25, 50, 100, and 150 ppm KMS in sugarcane juice were also performedto enhance preservation efficacy, with improved flavor, appearance, and overall acceptability during storage. 

Among the different sulphitation treatments, juices with 100 and 150 ppm KMS scored a high sensory grade (Table 2), which corroborated those evaluated by Bhupender et al. [27], and Rawat and Pohhriyal [28]. Among the sugarcane juices having a low pH, the juice with 4.25 and 4.5 pH revealed a significant reduction in SPC, favoring their preservation efficacy (Table 3).

### 3.2. Microbial Profile of Sugarcane Juice

**Figure 1:**
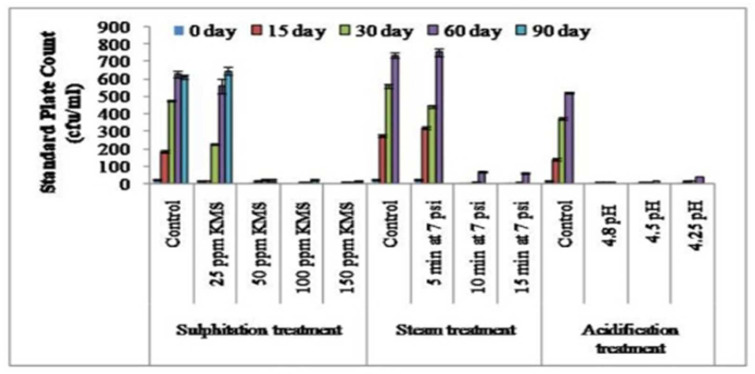
Effect of sulphitation, steam treatment, and acidification on standard plate count during the storage of sugarcane juice at refrigerator temperature.

**Figure 2:**
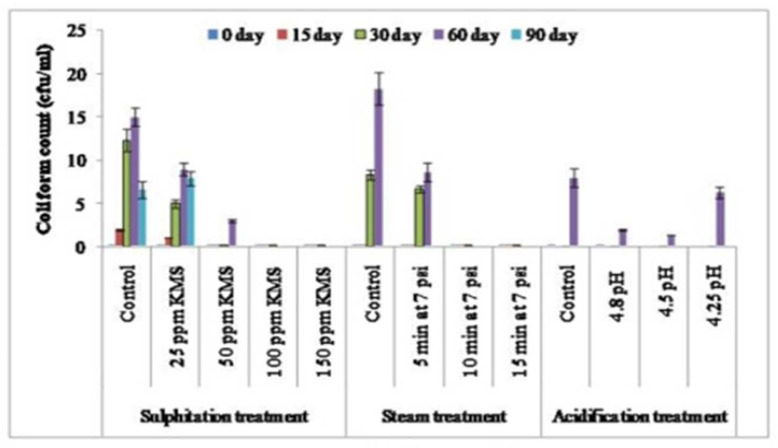
Effect of sulphitation, steam treatment, and acidification on thecoliform countduring storage of sugarcane juice at refrigerator temperature.

**Figure 3:**
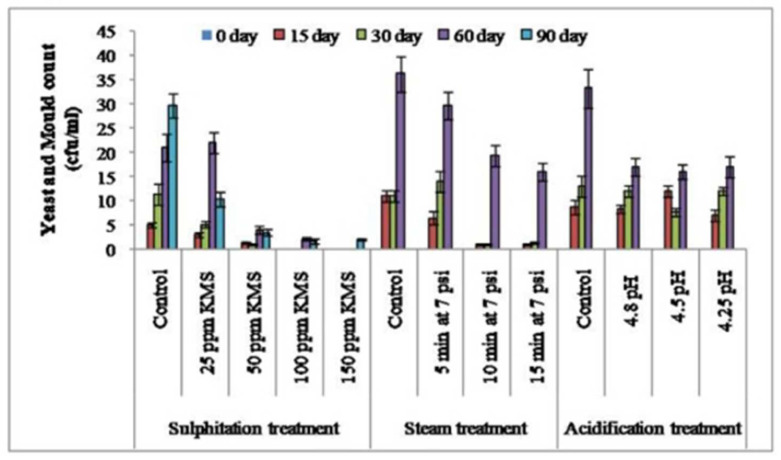
Effect of sulphitation, steam treatment, and acidification on yeast and mold count during storage of sugarcane juice at refrigerator temperature.

The microbial load, in terms of the SPC, coliform count, and yeast and mold count (cfu/mL) increased during the storage of sugarcane juice. In case of sulphitation (25, 50, 100, and 150 ppm KMS), the addition of KMS @ 100 ppm and 150 ppm restricted the growth of SPC (cfu/mL), the yeast and mold count (cfu/mL), and coliform count (cfu/mL) appreciably up to 90 days (Figure 1, Figure 2 and Figure 3). The studies reported by Khare et al. [16] showed similar outcomes, where sulphitation (KMS) at 225 ppm preserved the juice to 60 days in refrigerated conditions by arresting microbial growth. Hashmi et al. [29] also reported that mango pulp stored at ambient temperature (30–36 °C) with 0.2% KMS showed negligible microbial growth. Steam treatment for 10 and 15 min at 7 psi significantly limited the growth of SPC and the yeast and mold count (cfu/mL), and no coliform count (cfu/mL) was recorded compared to the control juice sample with up to 30 days of storage (Figure 1, Figure 2 and Figure 3). The results showed that effect of steaming at 80–85 °C for 5 and 10 min could decrease the microbial content and maintain the product properties [30,31,32]. However, with acidification treatment (4.25, 4.5, and 4.8 pH), the microbial profile investigated after 30 days of storage showed that the SPC (cfu/mL) was significantly less in acidified juice at pH 4.5, with a lower coliform count, while the yeast and mold counts were on par with the control and other acidified samples (4.25 and 4.8 pH). Chauhan et al. [26] also reported that adding citric acid (40 mg) and potassium metabisulphite (150 ppm) inhibited the growth of microorganisms and had a preservative action in sugarcane juice. Oladipo et al. [33] and Oranusi et al. [34] showed that when microorganisms are in acidic medium their growth rate was reduced, but as the pH tends from an acidic to basic medium; the growth rate of all the microorganisms increased in juice, which shows that the acidic medium greatly reduced their growth, while in a basic medium their growth was favored. The extent of increase in microbial counts followed this order: control sample > acidification (4.8, 4.5 and 4.25 pH) > steam treatment (10 and 15 min at 7 psi) > sulphitation (100 and 150 ppm KMS).

### 3.3. Physico-Chemical Parameters

#### 3.3.1. Titrable Acidity (% Citric Acid) and pH

Spoilage of juice is a functional result of fermentation and results in the deterioration of the product [27]. The results presented in Table 1 show that the titrable acidity increased significantly and the pH decreased in juice samples, including the control (up to 165.88%), 25 ppm KMS (162.75%), and 50 ppm KMS (124.07); while in the 100 ppm KMS and 150 ppm KMS treatment, a negligible change was noticed in the acidity and pH during a storage period of 90 days. These results are in accordance with the results of Chauhan et al. [27]. Similar studies conducted by Nisar et al. [35] reported combined impact of 0.1% KMS + 0.1% citric acid embedded with pasteurization (65 °C for 30 min) and stated that mango pulp had extended shelf stability for 90 days at ambient temperature (25 °C). This may have been due to management of antioxidant activity, which prevented browning. The addition of 100 ppm potassium metabisulphite maintained the pH and titrable acidity and preserved lemon juice at refrigerator temperature for 90 days [17]. In the case of steam treatment for 10 and 15 min at 7 psi, the pH and titrable acidity of the stored juice could be maintained only up to 30 days (Table 2). This may have been due to the restriction of microbial activity (Figure 1, Figure 2 and Figure 3) in the stored juice from thesteam treatment. It may alsohave been be due to the management of antioxidant activity, which prevented browning [15]. In the acidification treatment, theaddition of citric acid resulted in increasing the acidity and decreasing the pH during storage, thereby lowering the activity of yeast and mold.

#### 3.3.2. Total Soluble Solids, Total Sugar, and Reducing Sugars (%)

As we can see from Table 1, a significant increase in ˚Brix and total sugar (up to 15%) was observed with the increase in KMS concentration, whereas the reducing sugar content increased only upto 25 ppm KMS; thereafter, it decreased in the juice during 90 days of storage. Similar results were also observed with the steam treatment and acidification (Table 2 and Table 3). Organoleptically, the bottled juice with 100 and 150 ppm KMS preservative (up to 90 days) and 10 and 15 min steam treated juice (up to 30 days) tasted sweeter during storage, probably due to the slow release of the fructose moiety of sucrose in the solution. The sensory quality of the juice with preservative remained unchanged during storage at these time regimes. The ˚Brix and % total sugar gradually decreased in the acidified juice atall tested pHs with a 30 day storage period. This decrease in ˚Brix and % total sugar was directly related with the microbial action on to the juice [27]. The reducing sugar levels in thesamples increased significantly during storage, due to the hydrolysis of non-reducing sugar [36]. The addition of KMS @ 100 and 150 ppm and steam treatment for 10 and 15 min at 7 psi reduced the reducing sugars, probably due to suppression of microbial activity.

#### 3.3.3. Total Phenolic Content (mg/g)

As per the available literature, gallic acid, coumaric acid, ferulic acid, caffeic acid, and chlorogenic acid contributed to thetotal polyphenolic profile of the sugarcane juice [37]. In our samples, the changes of total phenolic content during storage are shown in Table 1, Table 2 and Table 3 (gallic acid equivalent); where we observed that the phenolic content gradually decreased during storage in the control, 25 ppm KMS, and 50 ppm samples, but an increased amount of KMS (100 and 150 ppm) somehow captured this oxidation, and hence there was a non-significant reduction i.e., 3.38% and 1.88%, respectively, at 90 days of storage. These results are similar to those observed previously by Setyawati et al. [38], Maathumai et al. [39], and Utama et al. [40], where sodium sulfite inactivated polyphenol oxidase and reduced phenol oxidation, and hence this induce phenol accumulation in the tissue. Similarly, in the steam-treated samples, no change in phenolics was observed, and a non-significant reduction of phenolic content was seen during the storage of juice up to 30 days. The steaming process can cause the thermal degradation of phytochemicals but it can also increase their total content by enhancing their availability for extraction, inactivating the polyphenol oxidase, or releasing fiber-bound polyphenols into free polyphenols [41,42].

#### 3.3.4. Polyphenol Oxidase (PPO) Activity (unit/mL)

**Figure 4:**
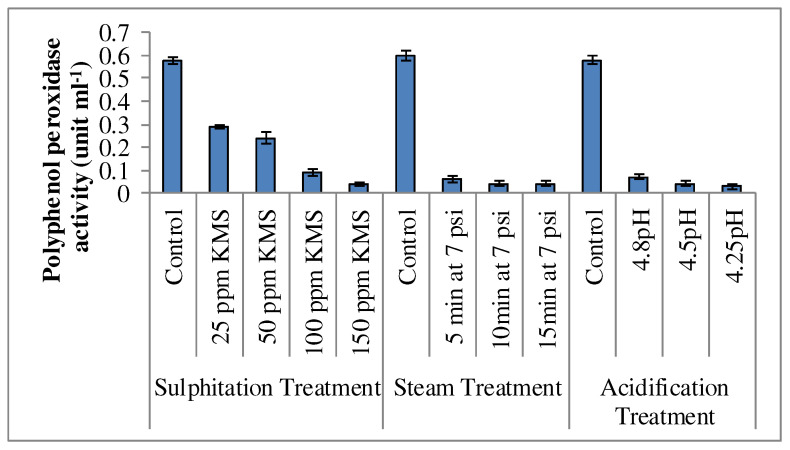
Effect of sulphitation, steam, and acidification treatments on the polyphenol oxidase activity (the numerical mean and the error bars (S.E) of triplicates (n = 3) are displayed in vertical bars).

The fresh sugarcane juice was anolive green color, which became brown during processing and storage in a refrigerator, and degreening appeared ata rapid rate with theincrease of the storage period. However, the steam, KMS, and citric acid treated sugarcane juice samples maintained their slight light green color. The color of the steam heated (5–15 min) and KMS (100 and 150 ppm) treated juice was stable up to 30 days and 90 days, respectively, in a refrigerator. The stability of juice color might have been due to theinactivation and reduction of PPO activity. The polyphenol oxidase activity was about (0.58 unit/mL) in the untreated juice (control), as shown in Figure 4. The addition of potassium metabisulphite (50–150 ppm), steam treatment (5–15 min), and acidification (4.8–4.25 pH) significantly reduced the PPO activity, which was negligible during storage. Polyphenol oxidase enzymes are destroyed at high temperature (80 °C), because they are heat labile [43]. Stream (121 °C/10 and 126 °C/10), citric acid (1% and 2%), and sodium metabisulphite (0.5%, 0.1% and 0.3%) treatments of sugarcane stalk have the capability to inactivate PPO enzymes and prevent the enzymatic browning caused by the PPO enzyme, which can enhance the shelf life of sugarcane juice [15].

#### 3.3.5. SO_2_ Concentration (ppm)

**Figure 5:**
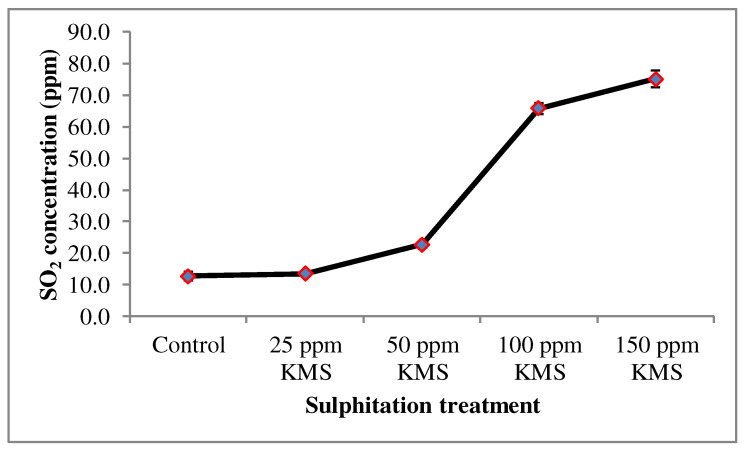
Effect of sulphitation on the SO_2_ concentration during refrigerator storage (the numerical mean and the error bars (S.E) of triplicates (n = 3) are displayed in the line graph).

As shown in Figure 5, a significantly higher SO_2_ concentration was recorded in 100 ppm and 150 ppm KMS treated juice than 25 ppm and 50 ppm KMS after 90 days of storage. The SO_2_ content released by potassium metabisulphite is an efficient antimicrobial agent, as well as an ascorbic acid stabilizer, which in turn depends on the pH of the juice [44].

### 3.4. Sensory Evaluation

The sugarcane juice, after preparation, was awarded sensory scores ranging between 8.0–8.5 for appearance, flavor, and overall acceptability by the semi-trained panelists. The sensory score reduced significantly with an increasing storage period. However, the reduction in sensory score was significantly greaterin the control, acidified, 25 ppm, and 50 ppm KMS treated juice treatments than in the steam treatments (10 and 15 min) and sulphited juice treatments (100 and 150 ppm). Eissa et al. [15] also reported that sodium metabisulphite (SO_2_), citric acid (CA), and thermal treatment inhibited the browning of sugarcane juice and maintained its green color up to four weeks at refrigerator temperature. SO_2_ and CA are considered anti-browning agents, by controlling the enzymatic browning reaction.

## 4. Conclusions

Briefly, among the selected set of treatments, the samples subjected to sulphitation @ 100 ppm and 150 ppm (under permissible range of KMS i.e., 700 ppm as per FSSAI) resulted in avery goodshelf stability forsugarcane juice, by arresting all physico-chemical and sensorial deterioration, and restricted the microbial proliferation up to 90 days in refrigerated conditions. Similarly, steaming cane juice samples (10 and 15 min at 7 psi), as a sole treatment, also proved to be an effective approach to impart an extension in preservation efficiency upto 30 days, without any preservative. Acidification (4.5 pH) with 50% citric acid could retard the microbial load for up to 30 days at refrigerator temperature but could not obtain a satisfactory sensory score. The applied hurdle technological concept, with cane steaming, sulphitation, and acidification, prevented the proliferation of micro flora to a greater extent than in the control juice, as well as preventing browning. Hence, it is proposed that the standardized treatments in our study could be used as an approach to extend the shelf life of sugarcane juice, with minimal sensory changes. 

## Figures and Tables

**Table 1:** Two-way ANOVA showing the effect of steam treatment on thephysico-chemical properties and sensory evaluation atdifferent storage intervals of sugarcane juice.

Source of Variation	Degrees of Freedom	Mean Sum of Square								
		˚Brix (%)	Total Sugars (%)	Reducing Sugars (%)	pH Value	Titrable Acidity (%)	Phenolic Content (mg/gm)	Color and Appearance	Flavor	Acceptability
Treatment (T)	3	3.757 **	1.311 **	0.0024	0.394 **	0.749 **	2.313 **	51.844 **	35.974 **	43.934 **
Storage Time (ST)	3	19.881 **	18.991 **	0.345 **	3.017 **	1.476 **	15.747 **	63.623 **	65.704 **	88.650 **
T × ST	9	0.825 **	0.128 *	0.008 **	0.078 **	0.175 **	1.476 **	8.983 **	10.226 **	11.178 **
**Treatment/Traits**		**˚Brix**	**Total Sugars**	**Reducing Sugars**	**pH Value**	**Titrable Acidity**	**Phenolic Content**	**Color and Appearance**	**Flavor**	**Acceptability**
Control		19.50 ^b^	18.63 ^b^	0.64 ^a^	4.75 ^b^	1.11 ^a^	2.41 ^b^	3.09 ^b^	2.86 ^b^	2.9 ^b^
5 min at 7 psi		19.68 ^b^	18.62 ^b^	0.68 ^a^	4.74 ^b^	1.17 ^a^	2.29 ^c^	3.13 ^b^	2.57 ^b^	2.79 ^b^
10 min at 7 psi		20.56 ^a^	19.11 ^a^	0.66 ^a^	5.05 ^a^	0.71 ^b^	3.11 ^a^	6.71 ^a^	5.68 ^a^	5.98 ^a^
15 min at 7 psi		20.54 ^a^	19.26 ^a^	0.66 ^a^	5.07 ^a^	0.70 ^b^	3.10 ^a^	6.72 ^a^	5.73 ^a^	6.03 ^a^
Storage time										
0 days		21.13 ^a^	19.92 ^a^	0.49 ^d^	5.36 ^a^	0.49 ^d^	3.85 ^a^	8.15 ^a^	7.43 ^a^	8.17 ^a^
15 days		20.78 ^a^	19.47 ^b^	0.55 ^c^	5.09 ^b^	0.88 ^c^	3.40 ^b^	4.67 ^b^	3.89 ^b^	4.18 ^b^
30 days		20.14 ^b^	19.15 ^c^	0.72 ^b^	4.97 ^b^	0.97 ^b^	2.38 ^c^	4.08 ^c^	3.68 ^b^	4.05 ^b^
60 days		18.24 ^c^	17.08 ^d^	0.87 ^a^	4.19 ^c^	1.34 ^a^	1.27 ^d^	2.75 ^d^	1.83 ^c^	1.60 ^c^
Coefficient of variation (%)		2.351	1.313	6.844	2.476	7.726	4.052	6.322	9.97	3.756

* denotes significant at 5% and ** denotes significant at 1% probability levels. Different alphabet superscripts in the treatment and storage time denote they were significantly different compared to the others (Tukey’s post hoc tests, *p* < 0.05).

**Table 2:** Two-way ANOVA showing the effect of sulphitation on the physico-chemical properties and sensory evaluation at different storage intervals of sugarcane juice.

Source of Variation	Degrees of Freedom	Mean Sum of Square								
		˚Brix (%)	Total Sugars (%)	Reducing Sugars (%)	pH Value	Titrable Acidity (%)	Phenolic Content (mg/gm)	Color and Appearance	Flavor	Acceptability
Treatment (T)	4	6.316 **	3.888 **	0.169 **	1.530 **	0.681 **	12.179 **	103.782 **	80.751 **	98.893 **
Storage Time (ST)	4	5.846 **	6.629 **	0.572 **	1.213 **	0.595 **	6.453 **	64.833 **	51.970 **	61.444 **
T × ST	16	1.152 **	0.915 **	0.042 **	0.180 **	0.099 **	1.519 **	9.949 **	7.157 **	7.660 *
**Treatment/Traits**		**˚Brix**	**Total Sugars**	**Reducing Sugars**	**pH Value**	**Titrable Acidity**	**Phenolic Content**	**Color and Appearance**	**Flavor**	**Acceptability**
Control		19.35 ^c^	17.37 ^d^	0.79 ^a^	4.55 ^c^	0.99 ^a^	2.08 ^c^	3.15 ^c^	2.79 ^c^	2.58 ^d^
25 ppm KMS		19.50 ^c^	17.42 ^d^	0.81 ^a^	4.64 ^c^	0.98 ^a^	2.18 ^c^	3.53 ^c^	3.07 ^c^	3.15 ^c^
50 ppm KMS		20.18 ^b^	17.75 ^c^	0.78 ^a^	4.93 ^b^	0.78 ^b^	3.34 ^b^	5.1 ^b^	4.41 ^b^	3.63 ^b^
100 ppm KMS		20.65 ^a^	18.26 ^b^	0.60 ^b^	5.21 ^a^	0.57 ^c^	3.92 ^a^	8.25 ^a^	7.6 ^a^	7.71 ^a^
150 ppm KMS		20.76 ^a^	18.51 ^a^	0.60 ^b^	5.25 ^a^	0.58 ^c^	3.88 ^a^	8.14 ^a^	7.51 ^a^	7.81 ^a^
Storage time										
0 days		20.70 ^a^	18.51 ^a^	0.52 ^e^	5.26 ^a^	0.53 ^e^	3.97 ^a^	8.51 ^a^	8.12 ^a^	8.43 ^a^
15 days		20.57 ^ab^	18.43 ^a^	0.56 ^d^	5.12 ^b^	0.63 ^d^	3.59 ^b^	6.55 ^b^	5.48 ^b^	5.07 ^b^
30 days		20.28 ^b^	18.01 ^b^	0.7 ^c^	4.93 ^c^	0.75 ^c^	2.77 ^c^	4.66 ^c^	4.30 ^c^	4.24 ^c^
60 days		19.63 ^c^	17.38 ^c^	0.78 ^b^	4.71 ^d^	0.93 ^b^	2.65 ^c^	4.22 ^d^	3.72 ^d^	3.65 ^d^
90 days		19.25 ^d^	16.98 ^d^	1.01 ^a^	4.57 ^d^	1.01 ^a^	2.45 ^d^	3.31 ^e^	3.67 ^d^	3.50 ^d^
Coefficient of variation (%)		1.59	0.98	5.14	2.96	7.78	3.83	5.30	10.42	9.26

* denotes significant at 5% and ** denotes significant at 1% probability levels. Different alphabet superscriptsforthe treatment and storage time denote they were significantly different compared to the others (Tukey’s post hoc tests, *p* < 0.05).

**Table 3:** Two-way ANOVA showing the effect of acidification on the physico-chemical properties and sensory evaluation at different storage intervals of sugarcane juice.

Source	Degree of Freedom	Mean Sum of Square								
		˚Brix (%)	Total Sugars (%)	Reducing Sugars (%)	pH Value	Titrable Acidity (%)	Phenolic Content (mg/gm)	Color and Appearance	Flavor	Acceptability
Treatment (T)	3	0.618 *	0.572 **	0.0059	1.576 **	1.989 **	6.842 **	0.704	1.542 **	0.952 **
Storage Time (ST)	3	15.270 **	20.239 **	1.630 **	1.659 **	2.889 **	3.503 **	74.454 **	76.172 **	89.724 **
T × ST	9	0.160	0.202	0.012	0.046	0.030	1.368 **	1.336 **	1.293 **	1.447 **
**Treatment/Traits**		**˚Brix**	**Total Sugars**	**Reducing Sugars**	**pH Value**	**Titrable Acidity**	**Phenolic Content**	**Color and Appearance**	**Flavor**	**Acceptability**
Control		19.69 ^b^	18.58	0.79	4.78 ^a^	0.91 ^c^	2.33 ^b^	3.40	3.7 ^a^	2.98
4.8 pH		19.90 ^ab^	19.01	0.75	4.48 ^ab^	1.47 ^b^	3.98 ^a^	3.12	3.01 ^b^	2.33
4.5 pH		20.01 ^ab^	19.02	0.79	4.14 ^bc^	1.7 ^a^	3.78 ^a^	2.81	3.05 ^b^	2.46
4.25 pH		20.23 ^a^	19.03	0.77	3.96 ^c^	1.84 ^a^	3.71 ^a^	3.08	2.92 ^b^	2.59
Storage time										
0 days		21.03 ^a^	20.02 ^a^	0.5 ^c^	4.76 ^a^	1.09 ^c^	4.02 ^a^	6.68 ^a^	6.81 ^a^	6.69 ^a^
15 days		20.59 ^b^	19.5 ^b^	0.58 ^bc^	4.47 ^ab^	1.24 ^c^	3.77 ^b^	2.92 ^b^	2.51 ^b^	1.33 ^b^
30 days		19.73 ^c^	19.08 ^c^	0.7 ^b^	4.28 ^bc^	1.41 ^b^	3.24 ^c^	1.56 ^c^	2.36 ^b^	1.18 ^b^
60 days		18.47 ^d^	17.05 ^d^	1.31 ^a^	3.87 ^c^	2.19 ^a^	2.80 ^d^	1.25 ^c^	1.0 ^c^	1.17 ^b^
Coefficient of variation (%)		2.109	1.891	15.55	9.574	9.415	8.538	15.351	13.604	12.590

* denotes significant at 5% and ** denotes significant at 1% probability levels. Different alphabet superscripts in the treatment and storage time denote they were significantly different compared to the others (Tukey’s post hoc tests, *p* < 0.05).

## Data Availability

The data presented in this study are available on request from thecorresponding author. The data are not publicly available due to restrictions.
